# Herbal NF-κB Inhibitors Sensitize Rituximab-Resistant B Lymphoma Cells to Complement-Mediated Cytolysis

**DOI:** 10.3389/fonc.2021.751904

**Published:** 2021-12-08

**Authors:** Xiaowen Ge, Yiqun Du, Jianfeng Chen, Na Zhu, Jiamei Yao, Xin Zhang, Na Wang, Yujing Sun, Feng Gao, Weiguo Hu, Yingyong Hou

**Affiliations:** ^1^ Department of Pathology, Zhongshan Hospital, Fudan University, Shanghai, China; ^2^ Fudan University Shanghai Cancer Center and Institutes of Biomedical Sciences, Shanghai Medical College, Fudan University, Shanghai, China; ^3^ Department of Oncology, Shanghai Medical College, Fudan University, Shanghai, China; ^4^ State Key Laboratory of Oncology, National Sun Yat-sen University, Guangzhou, China

**Keywords:** rituximab resistance, CD59, NF-κB, curcumin, perillyl alcohol

## Abstract

**Background:**

Drug resistance remains a serious challenge to rituximab therapy in B-NHL (B cell non-Hodgkin’s lymphoma). CDC (complement-dependent cytotoxicity) has been proposed as a major antitumor mechanism of rituximab, and direct abrogation of CD59 function partially restores rituximab sensitivity with high efficacy. However, universal blockade of CD59 may have deleterious effects on normal cells. Sp1 regulates constitutive CD59 expression, whereas NF-κB and CREB regulate inducible CD59 expression.

**Methods:**

Immunohistochemistry (IHC) assay was used to detect the expression levels of CD59 and other related molecules. Quantitative Real-time PCR (RT-PCR) analysis was used to explore the levels of transcripts in the original and resistant cells. We chose LY8 cells to test the effects of NF-κB and CBP/p300 inhibition on CD59 expression using flow cytometry (FACS). Immunoblotting analysis was employed to detect the effects of curcumin and POH. The *in vitro* and *in vivo* experiments were used to evaluate the toxicity and combined inhibitory effect on tumor cells of curcumin and POH.

**Results:**

We demonstrated that herbal (curcumin and perillyl alcohol) blockade of NF-κB specifically suppresses the expression of inducible CD59 but not CD20, thus sensitizing resistant cells to rituximab-mediated CDC. Moreover, activation of NF-κB and CREB is highly correlated with CD59 expression in B-NHL tissues.

**Conclusions:**

Our findings suggest the potential of CD59 expression as a predictor of therapeutic efficacy of NF-κB inhibitors in clinical application as well as the rationality of a NF-κB inhibitor-rituximab regimen in B-NHL therapy.

## Introduction

Rituximab is an anti-CD20 chimeric monoclonal antibody and represents a revolutionary advance in B-NHL (B cell non-Hodgkin’s lymphoma) treatment in combination with cyclophosphamide, doxorubicin, vincristine, and prednisone (R-CHOP). The main suspected mechanisms by which rituximab eliminates CD20-expressing cells include complement-dependent cytotoxicity (CDC), and antibody-dependent cellular cytotoxicity (ADCC) (1–3). Although rituximab greatly improves the clinical outcomes of B-NHL treatment, approximately 50% of patients are initially unresponsive, and the majority of patients eventually become resistant to further rituximab treatment, thus hampering the drug’s curative efficacy (4). Intrinsic unresponsiveness and acquired resistance to rituximab are not fully understood and remain a matter of debate.

Analyses of FcγRIIIa polymorphisms have clearly demonstrated that these polymorphisms are critical determinants of natural killer cell function, thereby demonstrating the importance of ADCC activity in determining the clinical efficacy of rituximab (5). In addition, numerous studies have reported the significance of rituximab mediated CDC (6–12). To overcome rituximab resistance, enhancing CDC activity is an important approach (1). The over-expression of mCRPs (membrane complement regulatory proteins), confers resistance to rituximab by inhibiting rituximab-induced complement activation (1). Adjuvant agents that directly abrogate the functions of mCRPs, such as CD46, CD55 and CD59, sensitize B-NHL cells to CDC, thus amplifying the therapeutic capacity of rituximab (13–16). CD59 appears to be the most effective mCRP protecting B-NHL cells from rituximab-mediated CDC (17). CD59 but not CD46 or CD55 is over-expressed in a multiple rituximab-treated mouse model xenografted with follicular lymphoma (FL) cells isolated from a patient (18). Moreover, in a clinical study of chronic lymphocytic leukemia (CLL), the expression of CD59 but not CD55 significantly increased in patients who failed to clear CLL cells from circulation after rituximab treatment (19). A bispecific antibody against CD20 and CD59 was demonstrated more potent than that against CD20 and CD55 in increasing the efficacy of antibody-based immunotherapy (20). We also observed that only CD59 is over-expressed and highly associated with resistance in B-NHL cells and the unresponsiveness of CLL cells to rituximab (13, 21). Therefore, CD59 is of interest as a potential drug target to combat rituximab resistance (13, 14, 16, 21, 22).

We have reported that the widely expressed transcription factor Sp1 is responsible for the constitutive expression of CD59, whereas NF-κB and CREB regulate the inducible expression of CD59 *via* connection of CBP/p300 (23). In addition, SOX2 upregulates CD59 transcription in the epithelial cancer stem cells (24), and is stabilized *via* PI3K/AKT signaling to develop the resistance to R-CHOP regimen in diffuse large B cell lymphoma (DLBCL) (25). In the process of TGF-β-induced epithelial–mesenchymal transition (EMT), CD59 is significantly upregulated by Smad3 to evade complement-mediated attack in metastasis (26). Further, we also found that the PKC signaling pathway is significantly activated in rituximab-resistant Burkitt’s lymphoma (BL) cells, and the application of the PKC inhibitor midostaurin could significantly enhance the pro-apoptotic effect of rituximab, leading to a significant therapeutic effect in tumor-bearing mouse models (27). Here, we demonstrate that NF-κB signaling axis is responsible for inducible CD59 expression in rituximab-resistant B-NHL cells and that inhibition of this axis can reduce CD59 expression and consequently sensitize rituximab-resistant B lymphoma cells to CDC effect.

## Materials and Methods

### Cell Lines and Culture

Human DLBCL OCI-LY8 cells (abbreviated as LY8) and human BL Raji cells were cultured in IMDM medium or RPMI 1640 media (Hyclone, Logan, UT), respectively, supplemented with 10% heat-inactivated fetal bovine serum, 100 U/mL penicillin and 100 mg/mL streptomycin (Gibco, Paisley, UK) at 37°C in an atmosphere of 5% CO_2_. We further generated rituximab-resistant Raji cells that were resistant to CDC induced by different concentrations of rituximab according to our previous procedure (13). These cells were termed Raji2, Raji4, Raji8, Raji16, and Raji32 because they could survive complement attack induced by rituximab (Roche, Basel, Switzerland) at concentrations of 2.0, 4.0, 8.0, 16.0, and 32.0 μg/mL, respectively, in the presence of 10% NHS.

### Reagents

FITC-conjugated anti-mouse IgG, anti-β-actin (C4) (sc-47778), anti-TFIIB (D-3) (sc-271736), anti-CD59 (H-7) (sc-133170), anti-CD46 (M177) (sc-52647), anti-CD55 (H-7) (sc-133220), goat-anti-rabbit IgG-HRP (sc-2004), goat-anti-mouse IgG-HRP (sc-2005), anti-p65 (F-6) (sc-8008), anti-phospho-NF-κB (RelA-S536) (AP0475), anti-p50 (E-10) (sc-8414), anti-cRel (B-6) (sc-6955), anti-Sp1 (E-3) (360759), anti-CREB-1 (24H4B) (sc-271), anti-phosphorylated CREB-1(Ser-133) (sc-101663), and anti-TP53 (DO-2) (sc-53394) antibodies were obtained from Santa Cruz Biotechnology (Dallas, TX). Anti-phosphorylated Sp1 (Thr-453) (ab59257) antibody was obtained from Abcam (Cambridge, MA). Anti-phosphorylated Sp1 (BS4755) antibodies were obtained from Bioworld Technology, Inc. (St. Louis Park, MN). Anti-acetyl-TP53 (Lys-373) (06-916) antibody was obtained from Millipore (Billerica, MA). FITC-conjugated mouse anti-human CD59 mAb (p282/H19) (555763) was obtained from BD Pharmingen (BD Pharmingen, San Diego, CA). FITC-conjugated AffiniPure goat anti-rabbit IgG (H-L) (305-095-003), AffiniPure F(ab)′_2_ Fragment Goat Anti-human IgG F(ab)′_2_ Fragment specific (107192) and AffiniPure F(ab)′_2_ Fragment Rabbit Anti-human IgM, FC5_u_ Fragment specific (110309) were obtained from Jackson ImmunoResearch Laboratories, Inc. (West Grove, PA).

Curcumin, the L-type calcium channel blocker POH, pyrrolidine dithiocarbamate (PDTC), (−)-epigallocatechin gallate (EGCG), parthenolide, 1,2-bis (2-amino-5-methylphenoxy) ethane-N,N,N′,N′-tetraacetic acid tetrakis (acetoxymethyl) ester (BAPTA-AM), calpain inhibitor I, calpain inhibitor II, BMS-345541, BAY 11-7082, C646, bortezomib, thalidomide, As_2_O_3_, and SN50 were obtained from Sigma-Aldrich (St. Louis, MO). IKK inhibitor VII, NEMO-binding domain binding peptide (NBD), casein kinase II inhibitor, caffeic acid phenethyl ester (CAPE), and W13 were purchased from Calbiochem Corp (La Jolla, CA). Dehydroxymethylepoxyquinomicin (DHMEQ) was kindly provided by Dr. K. Umezawa (Keio University, Yokohama, Japan). Dimethyl sulfoxide (DMSO) was used as the solvent for reagents that were insoluble in aqueous media. The above reagents were prepared as stock solutions according to the manufacturer’s instructions. Propidium iodide (PI) was obtained from Invitrogen (Carlsbad, CA). All pooled NHS used as a source of complement was obtained from 20 healthy persons and was aliquoted and stored at -80°C until use.

### Patients

A tissue microarray chip containing 131 DLCBL tissue samples was purchased from Alenabio, Inc. (Cat #2086, Xi’an, Shanxi, China), the distributor of US Biomax, Inc. (Rockville, MD). In addition, tumor tissues from 16 DLBCL patients were fixed with 4% neutral formaldehyde, then dehydrated and embedded in paraffin, and HE (hematoxylin-eosin) stained after sectioning. Another independent set comprising 26 B-NHL patients between 2015 and 2018 from Zhongshan Hospital. All 26 patients underwent at least 6 cycles of R-CHOP (rituximab plus cyclophosphamide, doxorubicin, vincristine, and prednisone) treatment and had specimens of tumor tissue before and after the treatment. Then the tissue microarrays (TMAs) were constructed and the use of samples was approved by the Ethics Committee of Zhongshan Hospital of Fudan University (B2018-073R). All specimens were assessed independently by 2 pathologists. All B-NHL were diagnosed according to the World Health Organization’s 2008 diagnostic criteria and 2016 revised standards. All DLBCL patients were followed up for 5 to 57 months. The follow-up was carried out on an outpatient basis or by telephone. The survival time of each patient was observed and recorded. Overall survival (OS) refers to the time from pathological diagnosis to death for any reason.

### Complement-Mediated Cytolysis

Cell viability was determined by PI staining as described previously (21). Briefly, 1.0×10^6^/mL cells were pretreated with vehicle or inhibitors for 48 hours, and medium containing 10% NHS as the source of complement and rituximab (16 or 32 μg/ml) was added to each well. The mixtures were incubated for 4 hours at 37°C and placed on ice to stop complement activation. After washing with 1% BSA/PBS, the harvested cells were incubated with PI (2 μg/mL) at room temperature for 15 minutes and immediately analyzed on a Cytomics FC 500 MPL (Beckman Coulter, Brea, California). The PI-negative control cells without any treatment were regarded as live cells. The percentage cell death was calculated using the following formula: cell death (%) = 100× [1 - (live cells in treated sample/live cells in untreated control)]. The percentage inhibitor-enhanced effect (%) on rituximab-mediated CDC was calculated as follows: cell death (%) = (% of dead cells in rituximab with inhibitor sample) - (% of dead cells in inhibitor alone sample).

### Immunohistochemistry (IHC) Staining

IHC staining was performed using the Bond Max Autostainer (Leica Microsystems, Buffalo Grove, IL). Antigen retrieval was conducted by incubating tissue sections in Bond Epitope Retrieval Solution 1 (ER1) at 100°C for 30 minutes. After retrieval, the sections were sequentially incubated with primary antibody for 25 minutes, secondary antibody for 15 minutes, and polymer for 25 minutes. Colorimetric development was performed by diaminobenzidine treatment for 10 minutes (Bond Polymer Refine Detection; Leica Microsystems, Buffalo Grove, IL). Finally, the sections were counterstained with hematoxylin, dehydrated, and mounted in Cytoseal XYL (Richard-Allan Scientific, Kalamazoo, MI). The color of the antibody staining in the tissue sections was observed by microscopy (Leica Microsystems, Buffalo Grove, IL).

The IHC score assessment was slightly modified according to previous studies (28, 29) and independently performed by two experienced pathologists. Briefly, the entire slide was evaluated, and 5 fields were chosen at random at 400× magnification. First, a proportion score was assigned, which represents the proportion of positively stained tumor cells (0, <5/100; 1, 5/100 to 33/100; 2, 34/100 to 66/100; 3, > 66/100). Next, an intensity score was assigned, which represents the average intensity of positive tumor cells [0, none; 1, weak (1+), 2, intermediate (2+); and 3, strong (3+)]. The proportion and intensity scores were then added to obtain a total score, which ranged from 0 to 6. A score of 0 to 3 was considered Low Expression, and a score of 4 to 6 was considered High Expression. Finally, a heat map was constructed using Cluster3.0 software according to the case amounts of each score, and the R and *P* values were calculated using SPSS software (version 19.0; SPSS Inc, Chicago, IL).

### Xenograft Studies

Female, 8-week-old Balb/C nude mice were purchased from Slac Laboratory Animal (Shanghai, China) and maintained under specific pathogen-free conditions. Mice were acclimated in house at least 7 days prior to use. To ensure the resistance of the Raji 32 cell line, cells were treated with 32.0 μg/mL rituximab and 10% NHS for 1 hour at 37°C before being transplanted into the nude mice; subsequently, a total of 1.5×10^7^ Raji 32 cells were suspended in 100 μL 50% Matrigel (BD Biosciences, San Jose, CA) diluted in serum-free culture medium and injected subcutaneously into the right flank of each BALB/c nude mouse. After xenotransplantation, mice were randomized into vehicle and treatment groups. In order to observe the synergistic effect of the drugs *in vitro* or *in vivo*, we used a slightly lower concentration compared to other references (30–35). Curcumin (20 mg/kg body weight) or POH (50 mg/kg body weight) diluted in olive oil was injected intraperitoneally daily on day 8 for a total of 12 days, and rituximab (4 mg/kg body weight) was injected intraperitoneally on day 9 on 3 occasions 4 days apart (Q4D). Tumor size was measured with calipers, and tumor volume was calculated by the following formula: (width)^2^ × length/2 (13, 36).

### Flow Cytometry

The expression levels of CD20 and CD59 were determined by immunofluorescence assay. Briefly, 1.0×10^6^/mL cells were pretreated with vehicle or various pharmaceutical inhibitors for 48 hours at concentrations lower than those that induced 5% cell death as indicated in **Figure 3** and **Supplementary Figure 1**. Subsequently, the cells (1.0×10^6^ cells/mL) were harvested for immunofluorescence analysis as described previously (13). To detect the expression of CD20, the respective primary antibodies and FITC-conjugated secondary goat anti-mouse IgG antibodies were employed; FITC-conjugated mouse anti-human CD59 antibody was directly used to detect CD59 expression. The stained cells were then analyzed by Cytomics FC 500 MPL (Beckman Coulter, Brea, California). Data were analyzed with Summit software and are expressed as the mean fluorescence intensity (MFI) in a histogram. Animal studies were approved by the Animal Ethics Committee at Shanghai Medical School, Fudan University.

### Quantitative RT-PCR

Total RNA was extracted using Trizol reagent (Invitrogen, Waltham, MA) according to the manufacturer’s instruction. Reverse transcription was performed using 1 μg of RNA and a Reverse Transcription System (Promega, Madison, WI) for Quantitative Real-time PCR (RT-PCR). The input cDNA was standardized and amplified for 45 cycles with SYBR Green Master Mix and gene-specific primers on an ABI Prism 7900HT machine (Applied Biosystems, Waltham, MA). The cycling parameters were 95°C for 2 minutes, followed by 45 cycles of 95°C for 10 seconds and 60°C for 30 seconds. The β-actin mRNA level was used as an internal normalization control, and samples were analyzed in triplicate. The primers for amplifying β-actin and CD59 transcripts T1-T8, T1-T4, T5 and T6-T8 have been reported previously (23).

### Western Blot Analysis

Total cellular proteins were isolated using a mammalian protein extraction reagent (Sigma), and nuclear extracts for detecting NF-κB, CREB, Sp1 and TP53 were prepared using a nuclear protein extraction kit (Active Motif, Carlsbad, CA) with a modified procedure described previously (23).

### Statistical Analysis

Statistical analysis was performed with SPSS (version 19.0; SPSS Inc, Chicago, IL). Pearson χ2 test or Fisher’s exact test was applied for categorical variables and the *t* test was applied for continuous variables. Survival curves were built by Kaplan-Meier method and compared by log-rank test. All *P* value was 2- tailed, and differences were considered significant at values of *P* < 0.05.

## Results

### CD59 Expression Level Is Associated With Therapeutic Effect of R-CHOP

**Figure 1:**
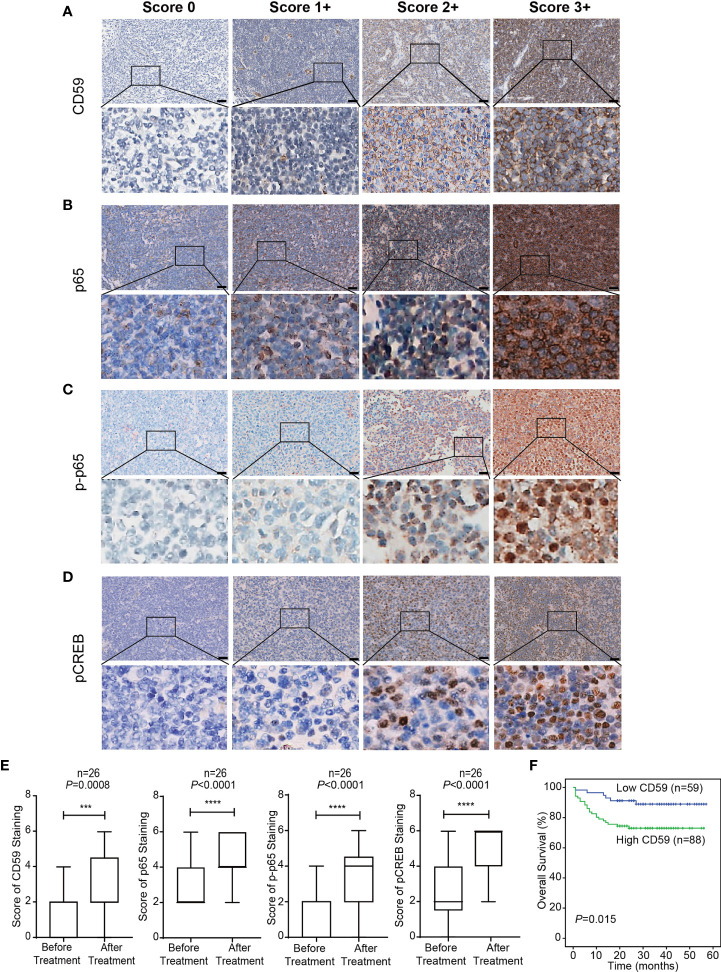
The expression level of CD59 in tumor tissues of DLBCL patients with R-CHOP treatment. **(A–D)** The representative Immunohistochemistry (IHC) images of CD59 **(A)**, NF-κB (p65) **(B)**, phosphorylated p65 (p-p65) **(C)** and phosphorylated CREB **(D)** staining. CD59 protein is mainly expressed in the cell membrane. NF-κB (p65) proteins is mainly expressed in the cytoplasm and nucleus. Phosphorylated p65 protein is mainly expressed in the nucleus. Phosphorylated CREB is mainly nuclear-localized. First column: score 0; Second column: 1+; Third column: 2+; Forth column: 3+; scale bar is 50 μm. **(E)** The expression of CD59 and NF-κB (p65), and the phosphorylation of p65 and CREB elevated significantly after R-CHOP treatment compared to the paired naïve DLBCL tissues. Values represent mean ± SD, n = 26. ***P < 0.001, ****P < 0.0001 *vs* before treatment, as analyzed by paired two-tailed Student’s *t* test. **(F)** IHC was used to detect the expression levels of CD59 in tumor tissues of 147 DLBCL patients. The Kaplan-Meier survival curve indicates that the high CD59 expression group has a shorter survival rate than the low CD59 expression group (χ^2^ = 5.875, *P*=0.015). A score of 0 to 3 was considered low CD59 expression, and a score of 4 to 6 was considered high CD59 expression.

IHC assay was used to detect the expression levels of CD59, NF-κB (p65), and the phosphorylation levels of p65 and CREB in 26 paired DLBCL patients before and after at least 6 cycles of R-CHOP treatment. The representative images of different IHC scores of CD59, NF-κB (p65), phosphorylated NF-κB (p65) (phosphorylated p65) and phosphorylated CREB staining are shown in **Figures 1A–D**, in which CD59 is mainly stained in the cell membrane. NF-κB (p65) protein is mainly expressed in the cytoplasm and nucleus. Phosphorylated p65 protein is mainly expressed in the nucleus, with inactive form in the cytoplasm, however, it has been reported that the increased expression of phospho-Ser536-p65 in the cytoplasm of the primary tumors correlated with worse survival of the patients independently of gender, age, tumor location, stage, and differentiation (37). Phosphorylated CREB is mainly nuclear-localized. We observed that the expression of CD59 (*P*<0.001) and p65 (*P*<0.0001), and the phosphorylation of CREB (*P*<0.0001) and p65 (*P*<0.0001) after R-CHOP treatment were significantly higher compared to the naïve treatment (**Figure 1E**). The expression levels of CD59 were also detected with IHC assay in another set of tumor tissues from 147 DLBCL patients, which were followed up for 5 to 57 months. The results indicated that the patients with high CD59 level had a shorter survival rate than those with low CD59 level (χ^2 =^ 5.875, *P*=0.015) (**Figure 1F**). Thus, in consistent with the previous reports (13, 14, 16, 21, 22), we further demonstrated that CD59 expression level may be a potential biomarker for DLBCL sensitivity to R-CHOP regimen.

### NF-κB and CREB Are the Major Trans-Acting Factors for Inducible CD59 Expression

**Figure 2:**
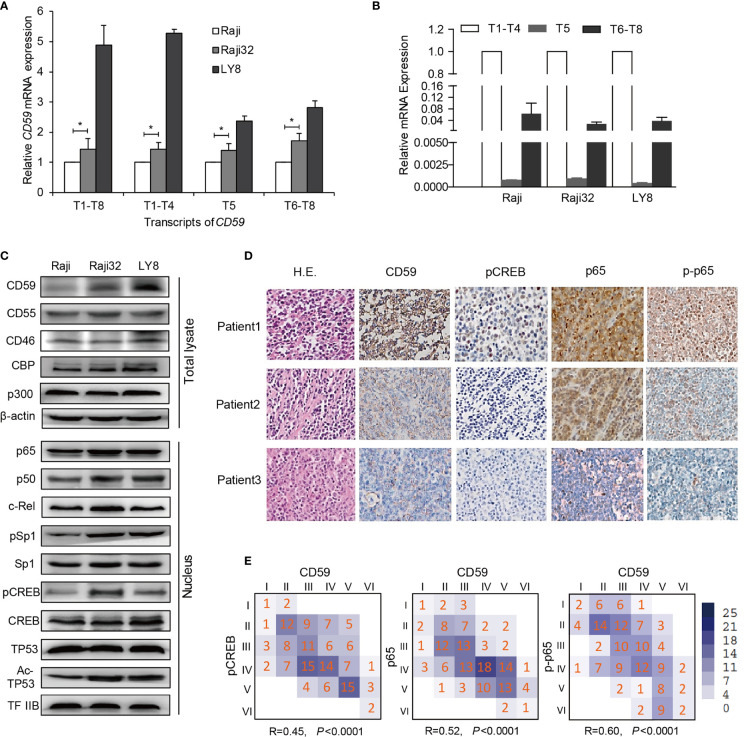
The inducible CD59 transcription is regulated by CREB and NF-κB. **(A)** A comparison of the abundance of *CD59* transcripts determined by qRT-PCR as the fold changes relative to Raji cells. Values are reported as mean ± SD of triplicate samples. *P < 0.05 vs. Raji, as analyzed by unpaired two-tailed Student’s *t* test. T1-T8: total transcripts; T1-T4: group 1 transcripts regulated by Sp1 or by NF-κB and CREB; T5: group 2 transcripts regulated by TP53; and T6-T8: group 3 transcripts regulated by CREB, as reported previously (23). **(B)** A comparison of the abundance of the three *CD59* transcriptional groups detected by qRT-PCR and normalized to T1-T4, n = 3. Values are reported as mean ± SD. **(C)** An immunoblotting assay was used to compare the expression levels of CD59 in total lysates and the nuclei of sensitive and resistant cells. β-actin and TFIIB were used as controls for the total lysate and nuclear fraction, respectively. **(D)** The IHC results of three representative DLBCL patients demonstrate that CD59 staining correlates well with the levels of NF-κB (p65), phosphorylated p65 (p-p65) and phosphorylated CREB. **(E)** Statistical correlation of CD59 expression with the activity or expression of its regulators. The Arabic numbers presented in the small squares represent case amounts, and the Roman numbers represent grade scores for each protein. The R and *P* values were calculated using SPSS software, n = 147.

We and others have previously demonstrated that the CD59-expressing subpopulation may be responsible for resistance to rituximab-mediated CDC activity (13, 18). Thus, we first generated five acquired rituximab-resistant Raji cell lines Raji2, Raji4, Raji8, Raji16, and Raji32 as described previously (13). The expression level of CD59 was closely correlated with the degree of resistance (**Supplementary Figure 1A**), whereas the expression levels of CD46, CD55 and CD20 were unchanged under the pressure of rituximab-mediated CDC (**Figure 2C** and **Supplementary Figure 1B**). We chose LY8 as the intrinsic rituximab-resistant cell line due to its high CD59 expression level (**Supplementary Figure 1A**). Functional tests revealed that the most resistant Raji 32 and LY8 cells were highly resistant to rituximab-mediated CDC compared to the original Raji cells, as indicated by the overwhelming PI staining in the majority of the original Raji cells (**Supplementary Figure 1C**).

Transcript 1-8 (T1-T8) indicate different transcripts of CD59 sharing a common open reading frame, which can be classified into three groups (T1-T4, T5, and T6-T8) based on their transcriptional initiation sites (23). T1-T4 are predominant and are regulated by Sp1 for constitutive CD59 expression or by NF-κB and CREB connected by CBP/p300 for inducible CD59 expression (23). Quantitative RT-PCR analysis revealed that the levels of all groups of CD59 transcripts were highest in LY8 cells and higher in Raji 32 cells than in original Raji cells (**Figure 2A**). As expected, the transcription levels of T1-T4 in LY8 cells were much higher than those of the other two groups of transcripts (**Figure 2B**). Furthermore, immunoblotting revealed corresponding CD59 translational levels, i.e., the resistant cells expressed higher levels of CD59 than rituximab-sensitive Raji cells. Moreover, the levels or activities of the relative trans-acting factors in the nucleus, including Sp1, NF-κB and pCREB, were higher in Raji 32 and LY8 cells than in Raji cells (**Figure 2C**). Together, these data indicate that NF-κB and CREB are the major trans-acting factors for higher inducible CD59 expression on resistant cells compared with sensitive cells.

IHC staining was also employed to determine the activation of signaling molecules that are relevant to CD59 expression. The IHC results of three representative DLBCL patients clearly demonstrate that CD59 staining correlates well with the levels of NF-κB (p65), phosphorylated p65 and phosphorylated CREB (**Figure 2D**). The statistical data for 147 DLBCL tissues further demonstrated that the IHC staining scores for p65, phosphorylated p65 and phosphorylated CREB are closely related to those for CD59 (**Figures 2D, E**; *P<*0.0001). Together, these results suggest that CD59 upregulation is mainly controlled by NF-κB and CREB in the acquired and intrinsic rituximab-resistant B cell lymphoma.

### NF-κB or CBP/p300 Inhibitor Suppressed CD59 Expression in a Dose-Dependent Manner

**Figure 3:**
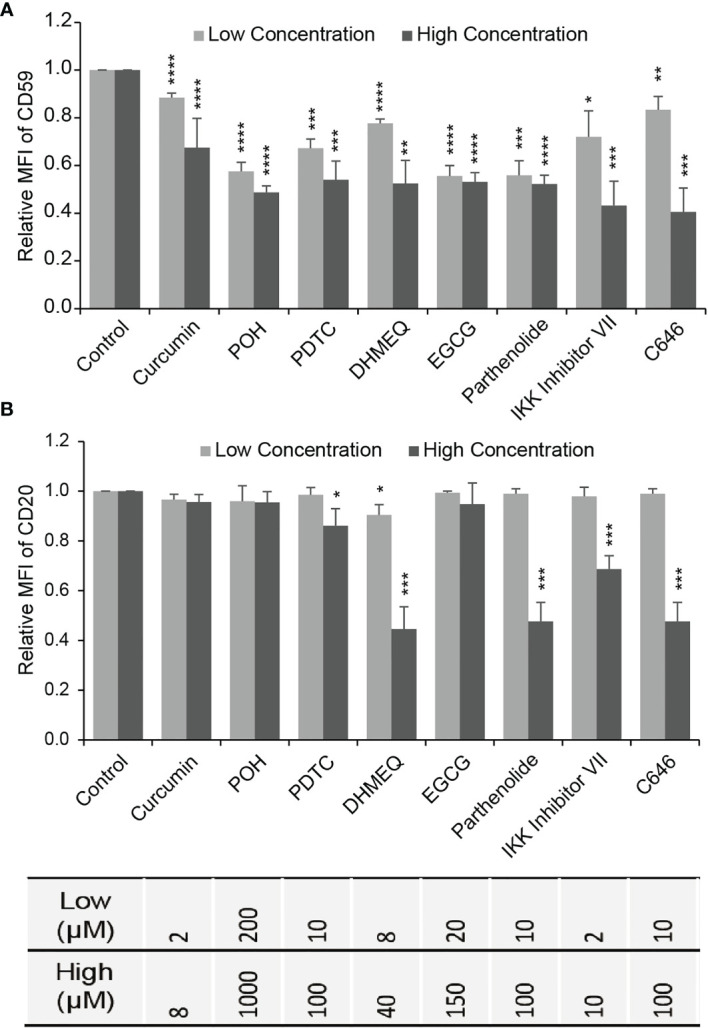
The effect of NF-κB or CBP/p300 inhibitor on the expression of CD59 and CD20. **(A, B)** The effects of NF-κB inhibitors (excluding C646) and CBP/p300 inhibitor C646 on CD59 **(A)** and CD20 **(B)** expression determined by FACS in LY8 cells. Gray bar: low concentration; Black bar: high concentration as indicated in **Supplementary Figure 2**. The values represent mean ± SD, n = 3. *P < 0.05, **P < 0.01, ***P < 0.001, ****P < 0.0001 vs. control.

We chose LY8 cells, which expressed the highest level of CD59, to test the effects of NF-κB and CBP/p300 inhibition on CD59 expression using flow cytometry (FACS). LY8 cells were treated with NF-κB inhibitors curcumin, POH (perillyl alcohol), BMS-345541, BAY 11-7082, DHMEQ, calpain Inhibitor I, calpain Inhibitor II, BAPTA-AM, W13, EGCG, CAPE, parthenolide, IKK Inhibitor VII, NBD, casein kinase II inhibitor, thalidomide, SN50, AS_2_O_3_, PDTC, or bortezomib, or CBP/p300 inhibitor C646 for 48 hours. FACS analysis revealed that, at inhibitor concentrations that induced less than 5% direct cell death, only curcumin, POH, PDTC, DHMEQ, EGCG, parthenolide, IKK inhibitor VII and C646 suppressed CD59 expression in a dose-dependent manner (**Figure 3A** and **Supplementary Figure 2**). Treatment with TNF-α but not B-cell activating factor (BAFF) increased CD59 expression, supporting our previous finding that the classical NF-κB pathway regulates CD59 transcription (23). However, the expression level of CD20 was reduced slightly by PDTC and was reduced significantly by DHMEQ, parthenolide, IKK inhibitor VII and C646, particularly at high doses (**Figure 3B**), which may compromise rituximab therapeutic effect (22). Therefore, three products from natural herbs, curcumin, POH and EGCG, appear to down-regulate specifically CD59 expression with no effect on CD20 expression, thus likely being potential for increasing rituximab-mediated CDC effect.

### The Herbal Products Curcumin and POH Down-Regulate CD59 Expression by Inhibiting NF-κB and CREB Activation

**Figure 4:**
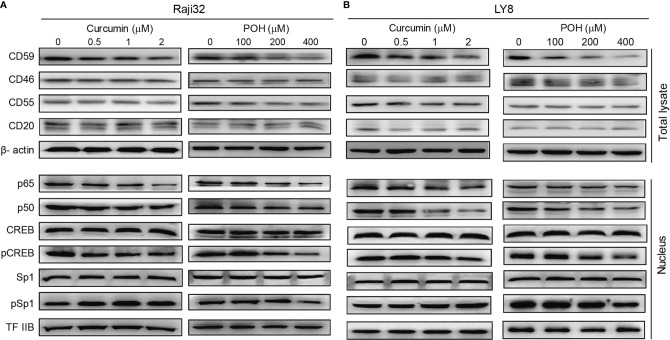
The herbal products curcumin and POH suppress CD59 expression *via* inhibition of NF-κB and CREB. **(A, B)** Curcumin or POH inhibits the activation of NF-κB and CREB but not Sp1 and further reduces CD59 but not CD46, CD55 or CD20 expression in Raji 32 **(A)** and LY8 **(B)** cells.

As mentioned above, three natural products, curcumin, POH and EGCG, suppressed the expression of CD59 but not CD20 in LY8 cells (**Figures 3A, B**), which strongly indicates these chemicals might be ideal adjuvants to relieve rituximab resistance. Therefore, we chose curcumin and POH to treat rituximab-resistant Raji 32 and LY8 cells. Immunoblotting analysis revealed that both curcumin and POH inhibited the phosphorylation of CREB and reduced the nuclear translocation of p65/p50, resulting in reduced expression of CD59 but not CD55, CD46 or CD20 in Raji 32 cells (**Figure 4A**). Similar results were observed in LY8 cells (**Figure 4B**). We did not observe any inhibitory effect of curcumin or POH on Sp1 expression or phosphorylation in either Raji 32 or LY8 cells (**Figures 4A, B**). Therefore, the natural compounds curcumin, POH and EGCG may compromise the expression of inducible CD59, but not constitutive CD59, CD46, CD55, and CD20 *via* inhibition of NF-κB and CREB.

### Curcumin and POH Sensitize Rituximab-Resistant B-NHL Cells to Rituximab-Mediated CDC

**Figure 5:**
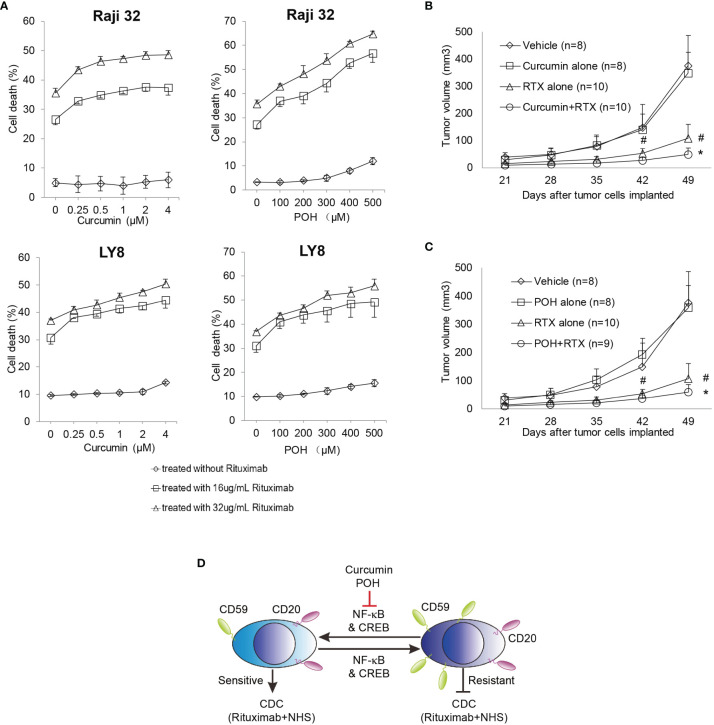
NF-κB inhibitors Curcumin and POH sensitizes the resistant Raji 32 and LY8 cells to rituximab-mediated CDC. **(A)** Raji 32 and LY8 cells were sensitized to rituximab-mediated CDC after pretreatment with curcumin or POH *in vitro*. The results represent mean ± SD, n = 3. **(B, C)** Curcumin **(B)** and POH **(C)** synergistically enhanced rituximab antitumor activity against resistant Raji 32 cells *in vivo*. One-way ANOVA followed by Tukey’s *post hoc* test was used to analyze *P* values. ^#^ *P* < 0.05 versus vehicle treatment. *P < 0.05 versus rituximab only treatment. The values represent mean ± SEM. **(D)** Schematic diagram of CD59 transcriptional regulation by NF-κB and CREB signaling. The natural chemicals curcumin and POH suppress the expression of CD59 but not CD20 *via* inhibition of NF-κB and CREB pathways, leading to sensitization of resistant Raji32 and LY8 cells to rituximab-mediated CDC. NHS, Normal Human Serum.

To eliminate a potential direct toxic effect of curcumin and POH, we minimized their administration dose in the *in vitro* and *in vivo* experiments. As shown in **Figures 5A–C**, curcumin or POH treatment alone at the given concentrations induced no or little direct toxicity in cultured or implanted Raji 32 and LY8 cells. In the *in vitro* experiments, the combination treatment of curcumin or POH with rituximab (16 and 32 μg/mL) and NHS increased rituximab-mediated CDC in Raji 32 and LY8 cells in a dose-dependent manner (**Figure 5A**). This synergetic effect of curcumin and POH was also observed *in vivo*. In the nude mice implanted with Raji 32 cells, we observed that the combination treatment of rituximab with curcumin or POH significantly slowed tumor growth compared with rituximab or POH treatment alone (**Figures 5B, C**). Unfortunately, we failed to subcutaneously implant LY8 cells in nude mice. Because the doses of curcumin and POH were selected to minimize the direct toxic effects of these molecules on implanted tumor cells, the administered doses likely did not achieve a therapeutic peak; therefore, the synergetic effect of enhancing rituximab-mediated *in vivo* CDC was not very impressive, although the data were statistically significant (*P*<0.05, rituximab alone *vs.* rituximab and curcumin or POH). Because curcumin and POH have little or no toxicity (34, 38) and may not affect Sp1 expression or subsequently alter constitutive CD59 expression, these findings strongly suggest that curcumin and POH are ideal adjuvants to restore rituximab sensitivity by suppressing the over-expression of CD59 on rituximab-resistant lymphoma cells. Therefore, our findings suggest that the combination treatment of NF-κB inhibitors and rituximab can enhance the therapeutic efficacy of rituximab by down-regulating CD59 expression and that CD59 is a potential biomarker for predicting the clinical application and therapeutic outcome of NF-κB inhibitors in B-NHL treatment (**Figure 5D**).

## Discussion

Curative treatment of and recovery from B-NHL remain challenging. Although the revolutionary development of rituximab has significantly improved the clinical outcome of B-NHL together with chemotherapy, intrinsic and acquired resistance to rituximab therapy compromise its use (1). Eventually, even patients who initially respond become more refractory to any available treatment. This chemo-immunotherapy is being strengthened by treatment based on novel targeted inhibitors such as bcl-2 inhibitor venetoclax or Bruton tyrosine kinase inhibitors, ibrutinib. However, the use of these agents may be associated with other disadvantages such as late side effects, problems with patient compliance, and selection of resistant clones (39). Herein, we identified another approach to conquering the resistance to rituximab-mediated CDC effect by reducing CD59 expression with herbal NF-κB Inhibitors.

CDC has been proposed as one of the major antitumor activities of rituximab, and thus a possible approach to combating rituximab resistance is enhancing CDC indirectly by inhibiting the functions of mCRPs, particularly CD59 (17). CD59 is constitutively expressed on almost all tissues, thus protecting autologous cells from complement attack (40); however, in lymphomas, CD59 is over-expressed to confer resistance to complement-mediated cytolysis (1). In the clinical B-NHL specimens, we observed that CD59 expression level elevates in B-NHL patients after the treatment of R-CHOP regimen, and high CD59 level predicts an unfavorable prognosis. Thus, it further supports that the functional abrogation of CD59 can significantly relieve rituximab resistance in B-NHL and CLL (13, 21, 41, 42). However, the universal inhibition of CD59 might eliminate the protective role of CD59 in normal tissue, resulting in deleterious side effects. We previously demonstrated that Sp1 regulates the constitutive expression of CD59, whereas NF-κB and CREB connected by CBP/p300 regulate inducible CD59 expression (20). Therefore, inhibiting only inducible CD59 expression in lymphoma cells but not constitutive CD59 expression in normal cells is an appropriate strategy to restore rituximab sensitivity.

Burkitt’s lymphoma (BL) is a highly aggressive lymphoma that represents up to 40% of lymphomas in children (43), although DLBCL is the most frequent type of B-NHL (44). Gene expression profiling has classified DLBCL into germinal center B cell-like (GCB) and activated B cell-like (ABC) subgroups based on cell-of-origin (45). In addition, compared with GCB DLBCLs, ABC malignancies more frequently exhibit constitutive activation of NF-κB (46). Thus, the cells lines Raji (BL) and LY8 (DLBCL/GCB) were used in this study to explore the inducible NF-κB activation and the downstream impact on CD59 expression. We observed a close relationship between inducible CD59 expression and the activity of NF-κB and CREB in above cell lines and clinical specimens.

Considering that the expression of CD20 is prerequisite for rituximab anti-tumor activity, the appropriate strategy for overcoming resistance should down-regulate the expression of only inducible CD59 but not CD20. We screened 22 agents that may inhibit NF-κB signaling, and found that some herb-derived products with no or little toxicity, including curcumin (47), POH (48), and EGCG (49), displayed such a potential. More importantly, in clinical trials, these natural products all exhibit varying levels of effects in patients with different cancers (50–53). Next, curcumin and POH were further tested whether they could restore rituximab sensitivity. We found that these two natural compounds could suppress the activation of both NF-κB and CREB, and then restrain the expression of inducible CD59 but not CD20, thus resulting in the *in vitro* and *in vivo* synergetic effect with rituximab in the resistant Raji32 an LY8 cells. Together, these findings warrant a clinical trial of a regimen comprising R-CHOP and natural NF-κB inhibitors such as curcumin and POH in B-NHL therapy.

## Data Availability Statement

The original contributions presented in the study are included in the article/**Supplementary Material**. Further inquiries can be directed to the corresponding authors.

## Ethics Statement

The studies involving human participants were reviewed and approved by Ethics Committee of Zhongshan Hospital of Fudan University (B2018-073R). Written informed consent for participation was not required for this study in accordance with the national legislation and the institutional requirements. The animal study was reviewed and approved by Animal Ethics Committee at Shanghai Medical School, Fudan University.

## Author Contributions

All authors contributed to the discussion of experimental design and data analysis. XG, YD, XZ, JC, NW, and YS did experiments. WH and YH conceptualized and designed the project, and wrote the manuscript. All authors contributed to the article and approved the submitted version.

## Funding

This work was supported by the National Natural Science Foundation of China (81372258, 81402570) to WH and XG; the Major State Basic Research Development Program of China (2013CB910802) to WH; the Program for Professor of Special Appointment (Eastern Scholar) at Shanghai Institutions of Higher Learning to WH; and Shanghai Municipal Key Clinical Specialty (shslczdzk01302) to YH.

## Conflict of Interest

The authors declare that the research was conducted in the absence of any commercial or financial relationships that could be construed as a potential conflict of interest.

## Publisher’s Note

All claims expressed in this article are solely those of the authors and do not necessarily represent those of their affiliated organizations, or those of the publisher, the editors and the reviewers. Any product that may be evaluated in this article, or claim that may be made by its manufacturer, is not guaranteed or endorsed by the publisher.

## Supplementary Material

Supplementary Figure 1 Evaluation of CD59 and CD20 expression on and cell death of sensitive Raji, acquired resistant Raji and intrinsic resistant LY8 cells during rituximab-mediated CDC by FACS. **(A, B)** Detection of CD59 **(A)** or CD20 **(B)** expression on original sensitive and acquired resistant Raji cells as well as intrinsic resistant LY8 cells. Cells were stained with anti-CD59 or anti-CD20 (black or purple lines as indicated) or an isotype-matched antibody (solid black lines), following by incubation with a FITC-conjugated secondary antibody. **(C)** Evaluation of cell death by PI staining. Solid black lines: untreated cells without PI staining; black lines: untreated cells with PI staining; and purple lines: cells treated with rituximab (2 μg/mL for Raji and 32 μg/mL for LY8 and Raji 32) and 10% NHS for 4 hours, followed by staining with PI. Representative data from 3 independent experiments are shown.

Supplementary Figure 2 Drug screening based on CD59 expression. The effects of NF-κB inhibitors (excluding C646) or CBP/p300 inhibitor C646 on CD59 expression in LY8 cells as determined by FACS. TNFα and BAFF were used to be control activators of canonical and non-canonical NF-κB pathway, respectively. The concentrations of different inhibitors are indicated in the table. The values represent mean ± SD, n = 3. **P* < 0.05, ***P* < 0.01,****P* < 0.001, *****P*<0.0001 vs. control.
