# Supplementary figures and images for: Herbal NF-κB Inhibitors Sensitize Rituximab-Resistant B Lymphoma Cells to Complement-Mediated Cytolysis
Source: Front Oncol. 2021 Dec 8;11:751904. doi: 10.3389/fonc.2021.751904 (PMC8692258; doi:10.3389/fonc.2021.751904)

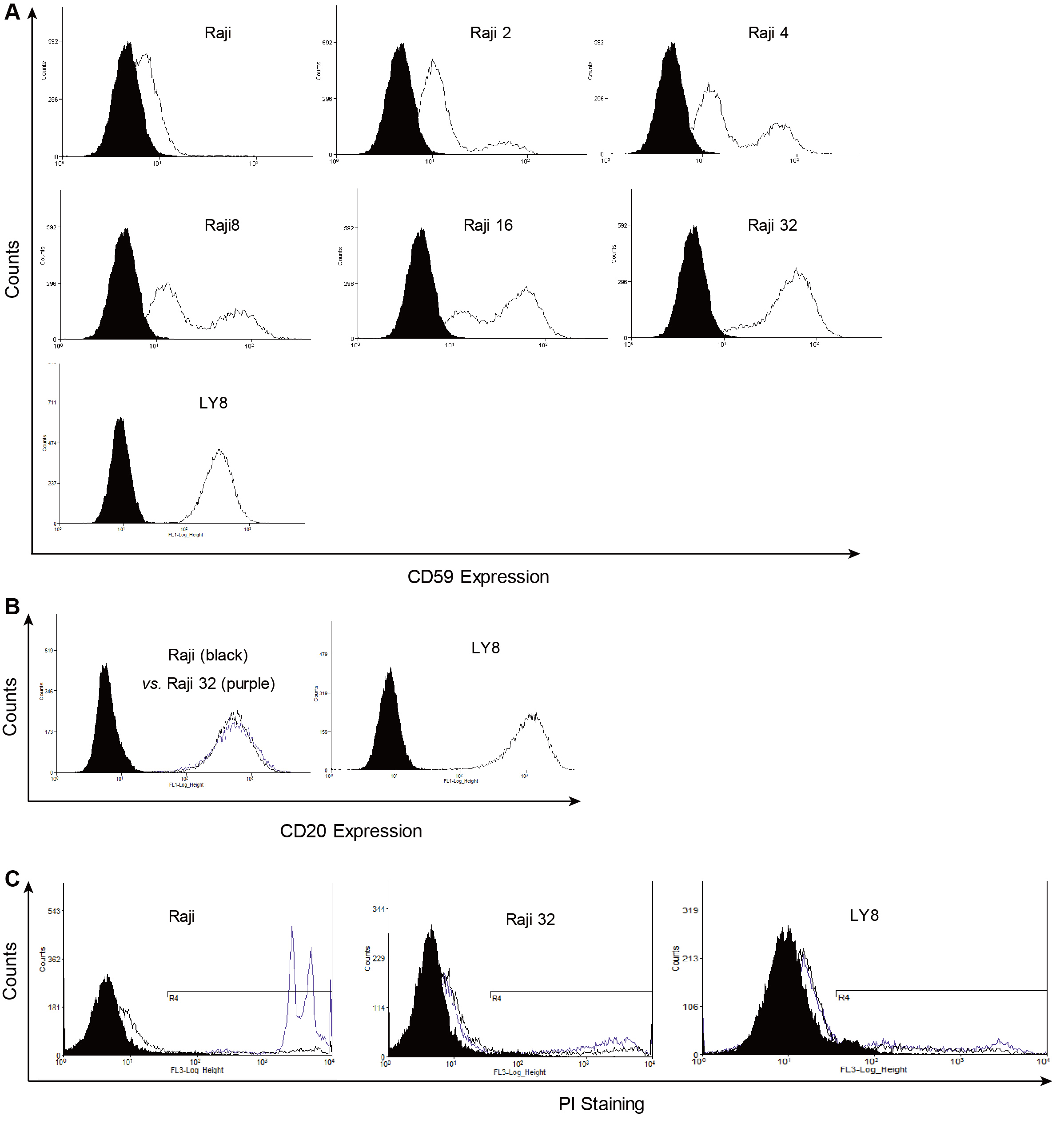

Supplement: Supplementary Figure 1 — Evaluation of CD59 and CD20 expression on and cell death of sensitive Raji, acquired resistant Raji and intrinsic resistant LY8 cells during rituximab-mediated CDC by FACS. (A, B) Detection of CD59 (A) or CD20 (B) expression on original sensitive and acquired resistant Raji cells as well as intrinsic resistant LY8 cells. Cells were stained with anti-CD59 or anti-CD20 (black or purple lines as indicated) or an isotype-matched antibody (solid black lines), following by incubation with a FITC-conjugated secondary antibody. (C) Evaluation of cell death by PI staining. Solid black lines: untreated cells without PI staining; black lines: untreated cells with PI staining; and purple lines: cells treated with rituximab (2 μg/mL for Raji and 32 μg/mL for LY8 and Raji 32) and 10% NHS for 4 hours, followed by staining with PI. Representative data from 3 independent experiments are shown. [file Image_1.jpeg]

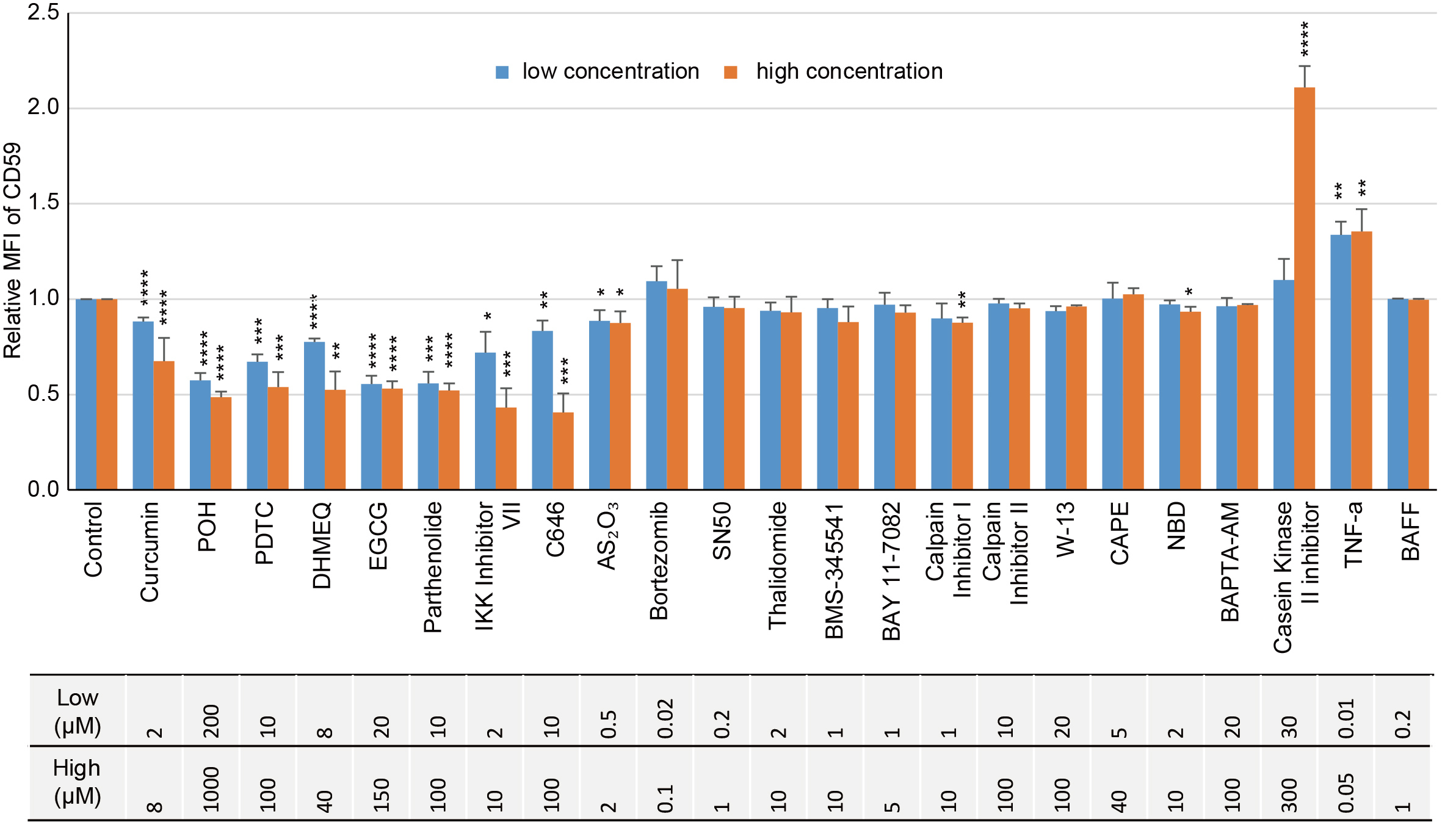

Supplement: Supplementary Figure 2 — Drug screening based on CD59 expression. The effects of NF-κB inhibitors (excluding C646) or CBP/p300 inhibitor C646 on CD59 expression in LY8 cells as determined by FACS. TNFα and BAFF were used to be control activators of canonical and non-canonical NF-κB pathway, respectively. The concentrations of different inhibitors are indicated in the table. The values represent mean ± SD, n = 3. *P < 0.05, **P < 0.01,***P < 0.001, ****P<0.0001 vs. control. [file Image_2.jpeg]
